# ASSOCIATION OF NEIGHBORHOOD CHARACTERISTICS AND RACE ON HEALTH STATUS AND HEALTH CARE USE FOR OLDER ADULTS

**DOI:** 10.1093/geroni/igae098.3198

**Published:** 2024-12-31

**Authors:** Mohammad Usama Toseef

**Affiliations:** Oakland University/Corewell Health, Farmington Hills, Michigan, United States

## Abstract

Research suggests that neighborhood characteristics are important social determinants of health. However, there is limited evidence that explores the interaction of race/ethnicity and neighborhood characteristics on healthcare use. We study this issue and specifically focus our analysis on how older Arab Americans use healthcare relative to their White counterparts. We conducted a retrospective study among patients 65 years and older (N = 112,056) who were tested for SARS-CoV-2 at the emergency departments of a large integrated midwestern healthcare system between March 1, 2020, and July 31, 2022. We used area deprivation index (ADI) quintiles that allows ranking of neighborhoods based on socioeconomic disadvantage. The outcomes for our analysis were infection with SARS-CoV-2, hospitalization, ED revisit 30 days and readmission 30 days. We estimated logistic regression models controlling for race/ethnicity, ADI and interaction of these two. We found that older Arab Americans in the 3rd and 4th quintiles were more likely than Whites to test positive for SARS-CoV-2. Arab Americans in the 4th quintile were more likely to be admitted as compared to Whites while no significant differences were observed in the other quintiles. Arab Americans in the 3rd quintile of ADI were more likely to have an ED revisit within 30 days and re-hospitalized within 30 days as compared to Whites. This shows how culture and perception of healthcare provision can play a role in the use of healthcare resource and policymakers and stakeholders may find it useful to focus on neighborhood characteristics as captured by ADI or similar measures.

